# Supplementary material for: Reconciling Mining with the Conservation of Cave Biodiversity: A Quantitative Baseline to Help Establish Conservation Priorities
Source: PLoS One. 2016 Dec 20;11(12):e0168348. doi: 10.1371/journal.pone.0168348 (PMC5173368; doi:10.1371/journal.pone.0168348)
Supplement: S1 Dataset — (ZIP) [file pone.0168348.s002.zip › Taxa/Serra Norte/SN_2013/N5S/Lista N5S-17.pdf]

CAVIDADE N5S-0017

Classe	Ordem	Fam/Outros	Gên/Outros	Espécie	S-U	U-S	Zon.
Arachnida	Acari	Argasidae	*Ornithodoros*	sp.1		X	
Arachnida	Acari	Ixodidae		jovem	X		E/P
Arachnida	Acari	Mesostigmata		jovem	X	X	P
Arachnida	Acari	Prostigmata		jovem		X	
Arachnida	Acari	Trombiculidae		sp.39	X		P
Arachnida	Acari	Trombidiformes		sp.49	X		P
Arachnida	Amblypygi	Phrynidae	*Heterophrynus*	*longicornis*	4		E
Arachnida	Araneae	Caponiidae	*Nops*	sp.2	X		P
Arachnida	Araneae	Filistatidae		sp.1	X	X	
Arachnida	Araneae	Gnaphosidae	*Camillina*	sp.1	X		E
Arachnida	Araneae	Mysmenidae		jovem	X		E
Arachnida	Araneae	Ochyroceratidae	*Ochyrocera*	sp.1	X		E/P
Arachnida	Araneae	Ochyroceratidae	*Speocera*	sp.1	X		E/P
Arachnida	Araneae	Pholcidae	*Mesabolivar*	*eberhard*		X	
Arachnida	Araneae	Pholcidae	*Mesabolivar*	sp.1	X		E/P
Arachnida	Araneae	Scytodidae	*Scytodes*	*itapevi*		1	
Arachnida	Araneae	Scytodidae	*Scytodes*	sp.1	25		E/P
Arachnida	Araneae	Theraphosidae	aff. *Holothele*	sp.1	11	X	E/P
Arachnida	Araneae	Theridiidae	*Theridion*	sp.1	X		E/P
Arachnida	Araneae	Theridiosomatidae	*Plato*	sp.1	X	X	E/P
Arachnida	Opiliones	Sclerosomatidae	*Prionostema*	sp.1	1		E
Arachnida	Pseudoscorpiones	Cheiridiidae		jovem	X		E
Arachnida	Pseudoscorpiones	Chernetidae	*Spelaeochernes*	sp.1	X		P
Arachnida	Pseudoscorpiones	Chthoniidae	*Pseudochthonius*	sp.1	X		P
Chilopoda	Scutigeromorpha	Pselliodidae	*Sphendononema*	jovem	2		E
Diplopoda	Polydesmida	Fuhrmannodesmidae		jovem		X	
Diplopoda	Polydesmida	Pyrgodesmidae		sp.1		X	
Insecta	Blattodea	Blaberidae		sp.3	2	X	E
Insecta	Blattodea	Blattidae		sp.5	9		E
Insecta	Blattodea	Polyphagidae		sp.1	9		E/P
Insecta	Coleoptera	Elateridae		sp.1	X		P
Insecta	Coleoptera	Hydrophilidae	Sphaeridiinae	sp.		X	
Insecta	Coleoptera	Hydrophilidae		sp.4	X		P
Insecta	Coleoptera	Scarabaeidae		sp.1	X		P
Insecta	Diptera	aff. Conopidae		sp.	X		E
Insecta	Diptera	Brachycera		sp.		X	
Insecta	Diptera	Chironomidae		sp.	X		P
Insecta	Diptera	Culicidae	*Culex*	*dolosus*	X	X	
Insecta	Diptera	Dolichopodidae		sp.	X		E/P
Insecta	Diptera	Mycetophilidae		sp.		X	
Insecta	Diptera	Nematocera		sp.		X	
Insecta	Diptera	Psychodidae	Phlebotominae	sp.	X	X	E/P
Insecta	Diptera	Psychodidae	*Telmatoscopus*	sp.	X	X	P
Insecta	Heteroptera	Reduviidae	Emesinae	sp.2	2		E
Insecta	Heteroptera	Reduviidae	Reduviinae	sp.	12	X	E/P
Insecta	Heteroptera	Reduviidae	Triatominae	sp.	1		P
Insecta	Heteroptera	Veliidae	*Microvelia*	jovem	X		P
Insecta	Homoptera	Cixiidae		jovem	X		P
Insecta	Hymenoptera	Braconidae		sp.3	X		P

Insecta	Hymenoptera	Diapriidae		sp.2	X		P
Insecta	Hymenoptera	Formicidae	*Brachymyrmex*	sp.1	X		E/P
Insecta	Hymenoptera	Formicidae	*Dolichoderus*	aff. *bispinosus*	X		P
Insecta	Hymenoptera	Formicidae	*Gnamptogenys*	sp.1	X		E
Insecta	Hymenoptera	Formicidae	*Pheidole*	sp.4	X		E
Insecta	Hymenoptera	Formicidae	*Solenopsis*	sp.1	X	X	E/P
Insecta	Isoptera	Termitidae	*Nasutitermes*	sp.	X		E
Insecta	Lepidoptera	Gelechioidea		sp.	X		P
Insecta	Lepidoptera	Tineoidea		sp.		X	
Insecta	Lepidoptera	Tineoidea		sp.1	X		E/P
Insecta	Lepidoptera	Tineoidea		sp.3	X		E/P
Insecta	Lepidoptera	Tineoidea		sp.4	X		P
Insecta	Neuroptera	Myrmeleontidae		jovem	X		E/P
Insecta	Orthoptera	Phalangopsidae	*Paraclodes*	sp.	5	X	E/P
Insecta	Orthoptera	Phalangopsidae	*Phalangopsis*	sp.	64	X	E/P
Insecta	Psocoptera	Psyllipsocidae	*Psocathropos*	sp.1	X		E
Insecta	Psocoptera	Psyllipsocidae	*Psyllipsocus*	sp.3	X		E
Insecta	Thysanura	Nicoletiidae		jovem	X		E
Amphibia	Anura	Leptodactylidae	*Leptodactylus*	cf. *vastus*		X	
Amphibia	Anura	Strabomantidae	*Pristimantis*	cf. *fenestratus*	6		P
Mammalia	Chiroptera	Emballonuridae	*Peropteryx*	*kappleri*	2		E/P
Mammalia	Chiroptera	Mormoopidae	*Pteronotus*	*parnellii*	100		E/P
Mammalia	Rodentia	Cricetidae	*Rhipidomys*	sp.		1	
Reptilia	Squamata	Boidae	*Epicrates*	sp.		1	
Reptilia	Squamata	Gekkonidae	*Thecadactylus*	sp.	1		P
